# Clinical Management With High-Frequency Ultrasound of Recurrent Submental Abscess Formation After Filler Placement: Bacterial Contamination or Immune-Mediated Adverse Event?

**DOI:** 10.7759/cureus.58878

**Published:** 2024-04-23

**Authors:** Andrada-Gabriela Nicola, Marius Octavian Pricop, Benito Ramos-Medina

**Affiliations:** 1 Oral and Maxillofacial Surgery, University of Medicine and Pharmacy Victor Babes, Timisoara, ROU; 2 Oral and Maxillofacial Surgery, Hospital Universitario Santa Lucia, Cartagena, ESP

**Keywords:** filler complication, ultrasound-guided, hyaluronidase, submental abcess, hyaluronic acid filler

## Abstract

The authors present a case of a 29-year-old female patient with a recurrent submental abscess formation after chin augmentation with highly reticulated hyaluronic acid filler. We evaluate the possible cause of this complication and the result after clinical management with ultrasound-guided injection of hyaluronidase. We highlight the prevention, assessment and treatment with real-time imaging of hyaluronidase injection in the affected area, as a predictable approach for both the patient and the physician.

## Introduction

Soft tissue augmentation by fillers has become popular for facial esthetic improvement, being associated with an increased risk of complication [1], with suboptimal outcomes. Even though the origin of these complications is not totally clear, one of the major hypotheses is an exacerbated immune response of the organism against foreign body material [2].

Apart from esthetic complications, as the injection of hyaluronic acid filler breaks the skin barrier, there is an increased risk of bacterial, mycobacterial, viral and fungal infections. Although uncommon, even abscess formation may occur after filler placement, and their treatment may be prolonged with repeated courses of antibiotics and surgical drainage. Removal of the filler material is essential to achieve a definitive solution to the problem [3].

Hyaluronidase, an enzyme that degrades hyaluronic acid, is typically administrated via subcutaneous "blind" injections, but this technique may not lead always to clinical improvement, and multiple treatments may be necessary. High-frequency ultrasound (HFUS) is a first-line tool to identify the filler location, assessing its size, depth and precise anatomical position [3]. Performing hyaluronidase injections under HFUS guidance is a highly accurate and efficient technique to maximize its clinical benefits [4].

## Case presentation

**Figure 1:**
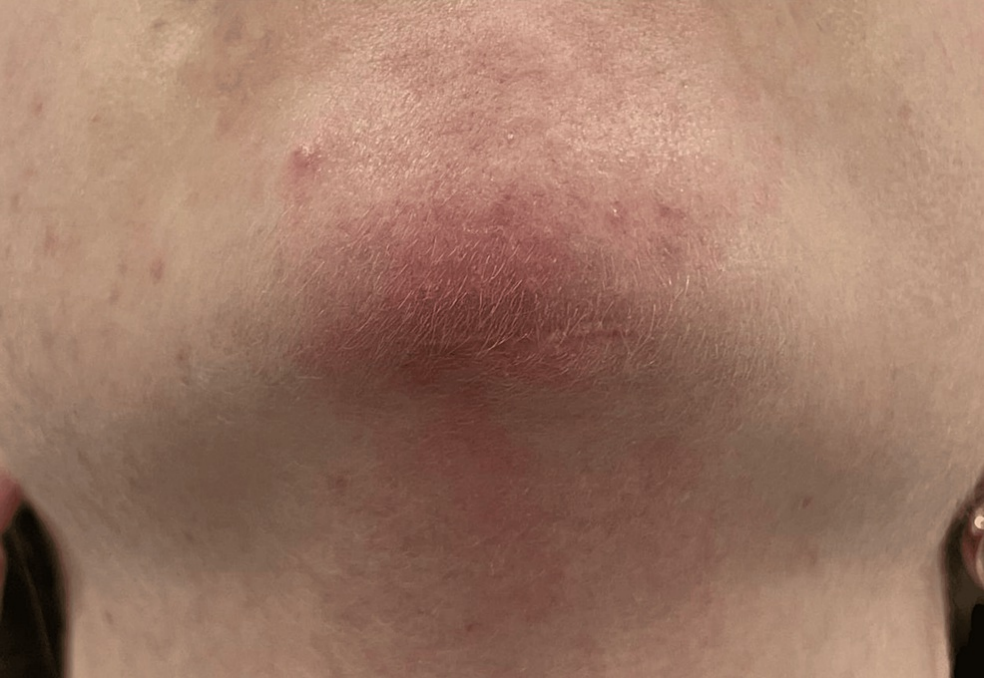
Initial examination revealing submental cellulitis and scarring from previous submental abscess drainages

A 29-year-old caucasian female patient presented to our outpatient clinic with a history of recurrent submental abscess following chin augmentation with highly reticulated hyaluronic acid filler one year ago, despite three surgical drainages associated with oral antibiotic therapy (amoxicillin, azithromycin, ciprofloxacin) and local "blind" injections of hyaluronidase. Clinical evaluation (Figure 1) revealed submental cellulitis including local pain, erythema, tenderness and swelling. 

**Figure 2:**
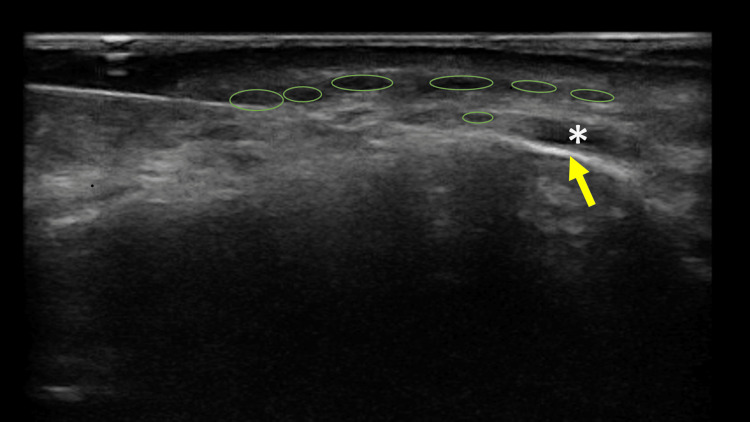
Submental high-frequency ultrasound exam revealing multiple well-defined millimetric oval-shaped anechoic deposits of residual hyaluronic acid filler (green circles). Mentalis muscle (white asterisk) and mandibular bone (yellow arrow) are identified.

Systemic therapy with antibiotics (clindamycin 300 mg three times a day, for 10 days) and corticosteroids (dexamethasone 4 mg, tapering method) was initiated. One week later, improvement of the local symptoms was noted. Following HFUS assessment (Clarius L20 HD3, 20 MHz, Vancouver, Canada), well-defined millimetric oval-shaped anechoic homogeneous deposits, were precisely identified in the subcutaneous and supra-periostal plane of the mental and submental region, corresponding to residual hyaluronic acid filler (Figure 2). 

**Figure 3:**
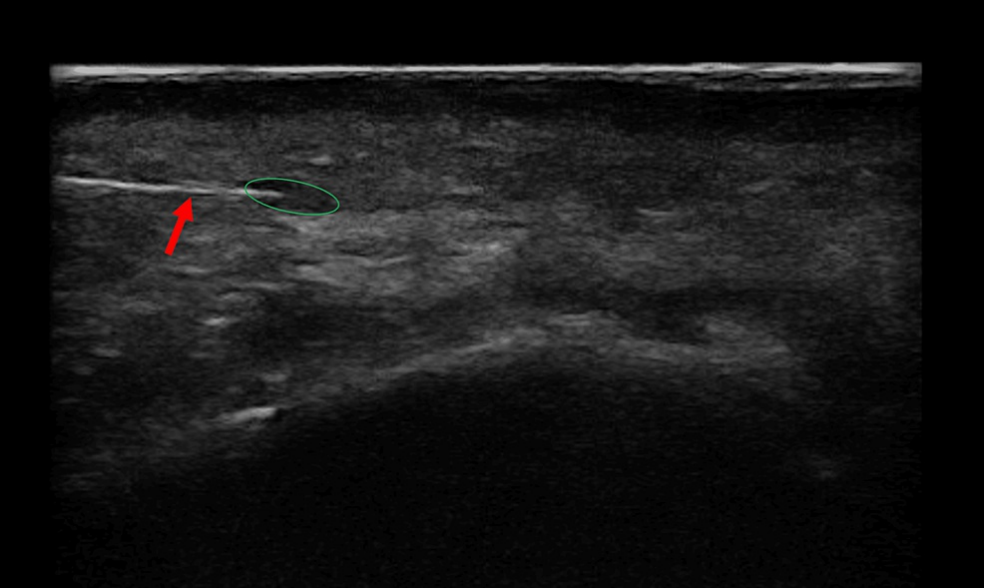
Precise ultrasound-guided injection of hyaluronidase. 27 G needle (red arrow) is clearly visible in the filler deposit (green circle).

**Figure 4:**
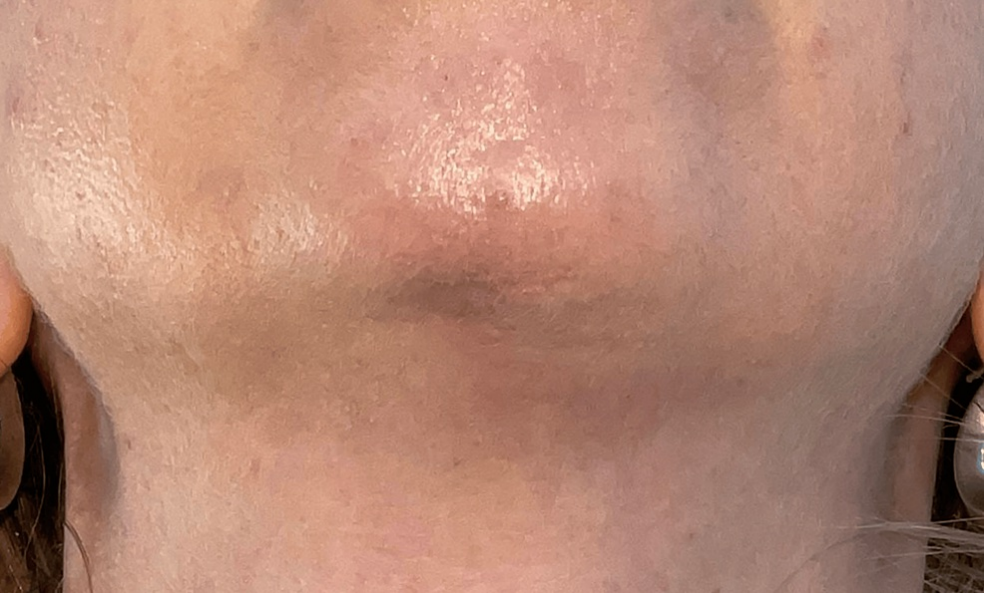
Complete healing of the submental region after treatment

Following skin disinfection and local anesthesia with bilateral mental nerve block, an amount of 30 IU of hyaluronidase (Skymedic, Barcelona, Spain) was injected under ultrasound guidance into each hyaluronic acid residual deposit (Figure 3), with a total of 280 IU used. Complete healing and stability of the result at one-year follow-up were achieved (Figure 4). 

Pus samples from previous drainages were provided by the patient, revealing no positive results for bacterial culture. However, this cannot exclude a technical error and contamination during the previous filler procedure. Furthermore, the formation of a sterile abscess raises questions regarding what triggered the inflammatory response. To detect if the hyaluronic acid filler by itself might be the trigger factor, we performed a genetic test (Fagron Genomics, Barcelona, Spain), which aimed to detect the existing combination of the human leukocyte antigen (HLA) subtype B*08* *and HLA subtype DRB1*03*, *that can increase the risk of developing immune-mediated rejection reactions to fillers [2]. DNA was obtained by oral swab, collecting buccal epithelial cells by scraping the inner side of the cheeks. The genetic filler test performed was negative, which does not completely exclude the risk of late-onset of side effects, but this probability is very low. 

## Discussion

With the increasing popularity of filler injections for cosmetic purposes, complications have become a hidden risk that can't be ignored, as these cosmetic procedures can affect the patient’s quality of life [1,5,6]. Recent researches focus on the evidence-based diagnosis and treatment of filler-based infections [3,4,7].

Similarly to other implants, dermal fillers carry the risk of biofilm formation, which can lead to the development of abscesses [8]. When a filler is injected into the skin, the biofilm (aggregate of encapsulated organisms in a polymeric matrix that adheres to the skin surface) can enter deeper structures and release bacteria to cause a local infection. Bacteria in biofilms have been detected in biopsies despite negative cultures. As biofilms progress, they become more resistant to antibiotics [3]. Biofilm-forming bacteria are generally opportunistic pathogens, such as the resident flora of the skin and mucosa (Streptococcus, Enterococcus, and Staphylococcus), considering the surface area of product (large bolus) and inadequate sterile technique, factors that have been hypothesized to play a role in biofilm development [8,9].

According to general guidelines for reduction of healthcare-associated infection, we highlight the importance of preparing the treatment area with adequate antiseptic substances for dermal filler injections, including 2% chlorhexidine gluconate in 70% isopropyl alcohol [9]. The operator should not only perform good disinfection but should also pay attention to sterility during the injection process [9]. Antibiotic therapy should be prescribed if signs or symptoms of acute bacterial infection are present [9]. The recommended initial empiric therapy includes amoxicillin/clavulanate 625 mg three times a day or clindamycin 600 mg three times a day for seven to 10 days [10].

On the other hand, some patients may experience immune-mediated reactions to fillers, as these substances are fundamentally considered foreign body materials. Decates et al. [2] show in their study that there is a link between specific HLA subtypes and an immune-mediated reaction to a foreign implant, meaning that some persons may be more prone to display a reaction than others, based on their immunogenetic profile. They found that people with an HLA* *subtype B*08 and HLA subtype DRB1*03* *combination have a 3.8-fold increase in risk of developing inflammatory adverse events due to fillers in clinical practice. In the present case, we performed this genetic filler test, taking into account the possibility of an immune-mediated response to the implanted material. 

Within the past 15 years, hyaluronidase has been used to dissolve cross-linked hyaluronic acid [11]. However, hyaluronidase should not be used in the presence of an active infection/cellulitis because it can facilitate the spread of infection into adjacent tissues. In our case, we do not inject hyaluronidase until initiating the antibiotic and corticosteroid therapy, waiting for the remission of the submental cellulitis. It is recommended that oral antibiotic treatment be provided for at least one week prior to hyaluronidase injection [3]. Related to biofilm formation, hyaluronidase will help break down the matrix, thus increasing the efficacy of antibiotic therapy, as well as in the case of a direct immunological reaction to the filler, hyaluronidase will remove the substrate [10].

Usually, hyaluronidase is injected by "blind injections" into a wide area around the injected filler, which is not a precise method, and small deposits of filler may still remain undissolved. In cases wherein undissolved fillers are still present despite several dissolution procedures, ultrasonography-guided hyaluronidase injection is a feasible and predictable approach [12].

Ultrasound is a non-invasive and helpful tool for diagnosis and management of complications. Pointing out the exact location of the cosmetic filler deposits allows for a safe procedure that benefits from direct visualization and guided injections [13,14]. We achieved a successful outcome by managing this complication with real-time ultrasound-guided hyaluronidase injections, with complete healing and no recurrence at one-year follow-up.

## Conclusions

To conclude, the case presented brings awareness to the clinician of the importance of pre-injection disinfection. Our case was unique in that the patient presented with recurrent submental abscess despite multiple surgical drainages, hyaluronidase injections and different antibiotic therapies. Appropriate management included the use of high-frequency ultrasound for diagnosing and guiding the hyaluronidase injection treatment. We strongly advocate this protocol in patients with hyaluronic acid filler complications, for a more precise, safe and effective medical approach.
